# Life-cycle mediated effects of urbanization on parasite communities in the estuarine fish, *Fundulus heteroclitus*

**DOI:** 10.1371/journal.pone.0225896

**Published:** 2019-12-02

**Authors:** James M. Alfieri, Tavis K. Anderson

**Affiliations:** 1 Ecology and Evolutionary Biology Interdisciplinary PhD Program, Texas A&M University, College Station, TX, United States of America; 2 Virus and Prion Research Unit, National Animal Disease Center, USDA—ARS, Ames, IA, United States of America; University of Potsdam, GERMANY

## Abstract

This study examined the relationship between urbanization and parasite community structure in the estuarine fish, *Fundulus heteroclitus*. We measured landscape and physicochemical factors associated with urbanization at 6 sites from 4 collection periods. Concurrently, we quantified the metazoan parasite community in *F*. *heteroclitus* collected at those sites, with 105 fish studied per site during the 4 collection periods. Parasite community composition differed among sites. Host size was the most important variable for direct life-cycle parasite assemblages and indirect life-cycle parasites at the individual fish level, while landscape and physicochemical factors determined the structure of indirect life-cycle parasite assemblages at the population scale. Variation in the prevalence and intensity of infection of two indirect life-cycle parasites, *Lasiocotus minutus* and *Glossocercus caribaensis*, were the primary parasites that drove differences across sites. Variation in the presence/absence of these indirect life-cycle parasite species was associated with sediment Ni concentrations, patch density, and marsh size. Our data support the hypothesis that urbanization, acting at both landscape and physicochemical scales, can have a significant impact on parasite community structure. This, however, varied by parasite life history: there was little effect of urbanization on the prevalence and intensity of direct life-cycle parasites, but significant variation was detected for indirect life-cycle parasites. This study demonstrates how anthropogenically driven landscape change influences fine-scale population dynamics of parasites.

## Introduction

Urbanization significantly alters estuaries and the free-living animal populations within these environments[1,2]. Animal parasites, which are intrinsically connected to their hosts, are also impacted by the consequences of urbanization. Urbanization can directly affect parasites through exposure to an altered physicochemical environment [3,4] or indirectly through changes in host population dynamics [5]. Parasites co-occurring in an individual–the parasite infracommunity [6]–simultaneously use the same host resource, but each parasite species may have a different life history strategy which may be differently affected by urbanization. Notably, indirect life-cycle (more than one host species) parasites have life stages that are exposed to the external environment and are dependent upon trophic interactions; whereas direct life-cycle (one host species) parasite transmission largely does not involve trophic interactions. Consequently, urbanization may differentially affect parasite species [7], but data that associates urbanization with parasite communities and life history strategy are sparse (but see [8,9]).

Parasite life-cycles can be broadly characterized as either indirect or direct. Indirect life-cycle parasites require different species of hosts to complete their life-cycle and are largely dependent on trophic interactions which occur more frequently in locally stable communities [10]. This essential interaction has been used to demonstrate how parasites can be indicators of free-living diversity [11], and as such, it has been argued that a healthy ecosystem is one that is rich in parasites [12]. In urbanized estuaries, host population dynamics may be less stable, resulting in reduced diversity or less predictable host dynamics, causing a concomitant decrease in the species richness of indirect life-cycle parasites [5]. On the other hand, direct life-cycle parasites require only one species of host to complete their life-cycle and are largely dependent on host population densities, as abundant hosts increase the probability of a parasite encountering a suitable environment to colonize [13,14]. A feature of urbanized estuaries is habitat fragmentation, which can result in increased host densities and a concomitant increase in the species richness of direct life-cycle parasites [15]. Generally, parasite communities are described using terms such as prevalence (the number of hosts infected with a parasite species divided by the number of hosts examined), and intensity of infection (number of individuals of a parasite species in a single host species) [6]. These measures are calculated by quantifying the parasite species in a host individual–the parasite infracommunity [6]–and then aggregating these data to consider all the parasite species–the parasite “component” community–found in a subset of a host species collected from a subset of a sampled abiotic environment [6].

Many of the studies that examine the interaction between host and parasite in urbanized ecosystems have been conducted with human pathogens [16], consider a single host and a single parasite [17], or a single contaminant and landscape factor. Further, linking the regional process of urbanization to local transmission and diversity of parasite communities has yet to reveal consistent patterns [18–20]. One potential explanation for the absence of consistent results is that urbanization differentially affects parasite species: the size and direction of effect is contingent upon parasite life history. In this study, we assessed the effect of multiple landscape composition and physicochemical factors on 24 parasite communities of the salt marsh fish, *Fundulus heteroclitus*. The parasite community in *F*. *heteroclitus* includes indirect and direct life-cycle species, and the impact of urbanization on the structure parasite communities should be mediated through different life-cycle strategies. We predicted that urbanized sites would have low diversity and prevalence of indirect life-cycle parasites; but direct life-cycle parasites would increase in diversity and abundance.

## Materials and methods

### Ethics statement

Field collections were conducted under scientific permits issued by the Georgia Department of Natural Resources approved collection under Scientific Collecting Permit number 29-WJH-15-182. Prior to necropsy, fish were maintained briefly in aquaria following animal care protocols approved by The Animal Care and Use Committee at Georgia Southern University (protocol #I14013). Fish euthanasia was conducted in accordance with the 2000 Report of the American Veterinary Medical Association Panel on Euthanasia, approved by The Animal Care and Facilities Committee at Georgia Southern University under the protocol described above (protocol #I14013): fish were placed in a buffered 300 mg/L solution of tricaine methanesulfonate (MS-222) until cessation of opercula movement, followed by pithing of the brain and spinal cord.

### Study site description

**Fig 1:**
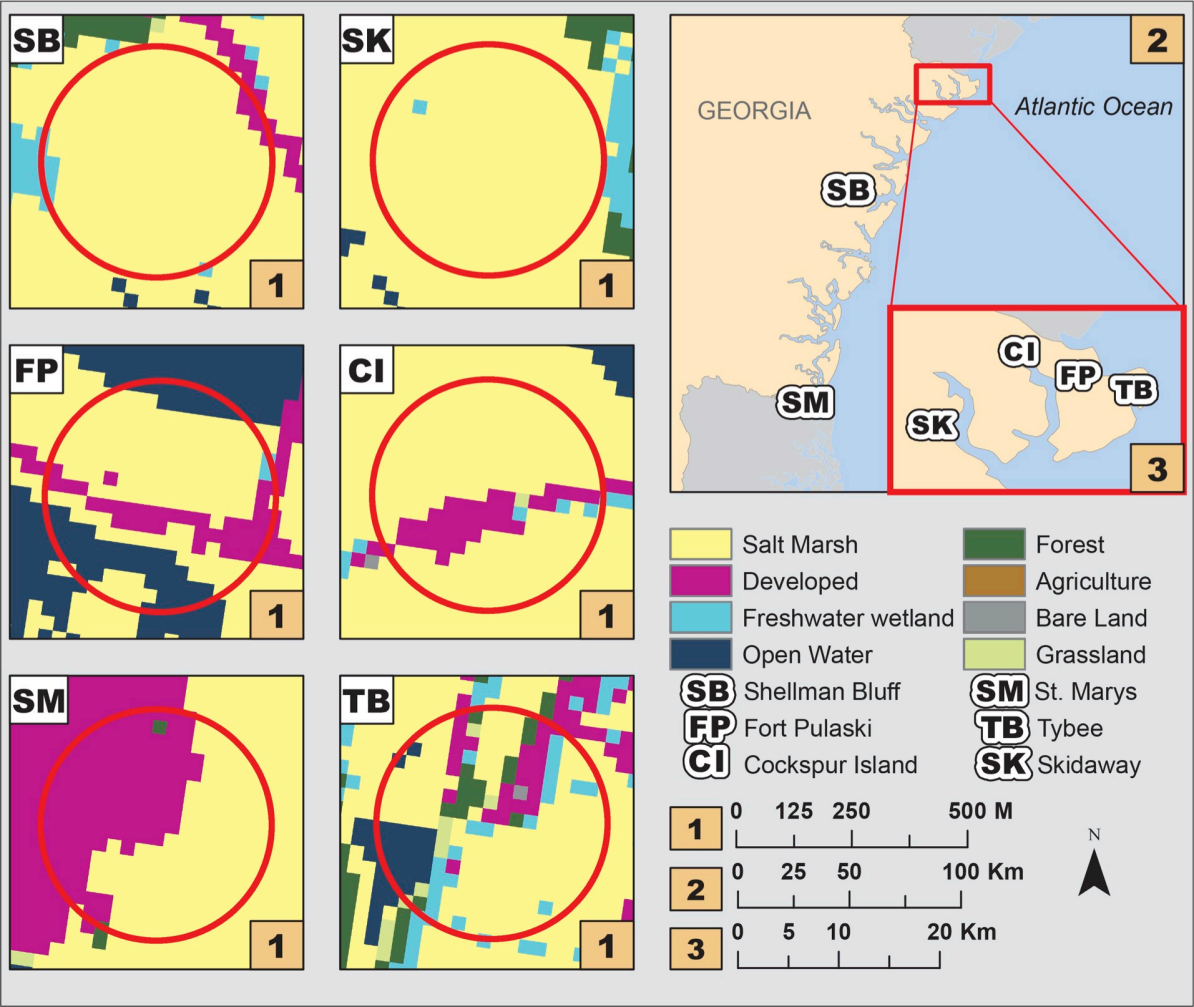
Map of 6 salt marsh study sites in coastal Georgia, USA with 2011 land cover data. The sites include Fort Pulaski (FP), Cockspur Island (CI), Tybee (TB), and Skidaway Island (SK) in the Savannah River Estuary; Shellman Bluff (SB) as a representative of the Sapelo Sound; and St. Marys (SM) as part of the St. Marys Estuary. Red circles represent a 250 m buffer around the study site. For the purpose of this work, land cover data was reclassified from the original 21 categories to 10 (Salt Marsh, Developed, Freshwater Wetland, Open Water, Forest, Agriculture, Bare Land, and Grassland): 2 land cover categories were not present in the study area (Freshwater Aquatic Bed and Unconsolidated Shore).

We examined 6 sites along the coastline of Georgia, USA, that reflected variation in urbanization, measured through % impervious surface within a 250 m radius around each site (Fig 1). We used this metric to select sites because impervious surface is a strong predictor of different effects of urbanization, such as landscape composition changes, habitat fragmentation, and contaminants [2,21]. The 250 m radius around each site was chosen because this is similar to the home range size of *F*. *heteroclitus* [22,23]. The 250 m radius criteria was used as an objective measure to select sites representing a range of urbanization and its effects, however, for the landscape composition analyses we used the watershed level as our spatial scale (described below).

### Quantifying the landscape and physicochemical environment

We defined the boundaries of the landscape to the lowest level watershed provided by the United States Geological Survey (USGS) Watershed Boundary Dataset (WBD): the hydrological unit code (HUC) 12-digit boundary [24]. We used the 2011 National Land Cover Dataset (NLCD) and National Oceanic and Atmospheric Administration’s (NOAA) Coastal Change Analysis Program (C-CAP) for the regional land cover analysis [25,26]. Regional landscape composition and change were measured using ArcGIS (ArcGIS Version 10.3, Environmental Systems Research Institute, Redlands, California). We also calculated a series of regional landscape metrics: patch density, largest patch index, edge density, mean salt marsh size, % salt marsh, % developed land, and total area using FRAGSTATS software [27]. Patch density is an index that expresses the number of continuous habitat types, or patches, per unit area, and is a metric for landscape heterogeneity. Largest patch index is the size of the largest habitat patch relative to the size of the landscape, and is a measure of landscape dominance. Edge density is an index of the amount of habitat edges per unit area, and the contrast between developed land and salt marsh was weighted at 0.5 to evaluate salt marsh habitat fragmentation by urban structures or landscapes. Mean salt marsh size is an index of the size of a salt marsh habitat patch, with larger patches weighted in order to decrease the sensitivity of the mean to small habitat patch sizes. The % salt marsh and developed land is the percentage of the landscape comprised of those categories, and is a metric for habitat size and dominance. Total area refers to the area of the watershed. In addition, we used the National Oceanic and Atmospheric Administration’s (NOAA) Coastal Change Analysis Program (C-CAP) to evaluate % landscape change within the HUC-12 digit watersheds for the period of time from 1996–2010.

We collected information on the physicochemical environment from each site at flow and ebb tide in March, June, September 2015 and February 2016. Flow and ebb tide measurements were averaged for each site and time point for analysis. We measured pH, temperature, salinity, and conductivity using a handheld multiparamenter instrument (Yellow Springs Instruments Professional Plus; Yellow Springs Instruments, Yellow Springs, Ohio). In addition, we quantified the trace metal composition, nutrient concentrations, and chlorophyll *a* concentration during these dates. To extract chlorophyll *a*, we followed a standard acidification method (EPA 445.0), whereby a volume of 100 ml was filtered using Whatman GF/F filters (47 mm; 0.7 μm nominal pore size). Phytoplankton pigments were then extracted from the filters in 8 ml of 90% acetone at 0°C for 24 hours. Fluorescence was measured using a Trilogy fluorometer (Turner Designs, San Jose, California). For the nutrient and trace metal quantification, 2 samples from each collection time and site were sent to the University of Georgia Laboratory for Environmental Analysis (Athens, Georgia). These samples were filtered using a microwave-assisted nitric acid method (EPA 3015), and total nitrogen, total phosphorus, and 72 metals were analyzed with inductively coupled plasma mass spectrometry (ELAN 9000; PerkinElmer, Waltham, Massachusetts). In addition to water analyses, we quantified the trace metal composition of sediment by collecting 3–8 cm core samples from randomized locations within each site in September 2015. These samples were sent to the University of Georgia Laboratory for Environmental Analysis (Athens, Georgia), and were prepared using a microwave-assisted nitric acid method (EPA 3052).

### Quantifying the parasite community in *F*. *heteroclitus*

We used the parasite community of a focal species, the common marsh killifish *F*. *heteroclitus*, to assess the impact of human modification of the environment on parasite establishment and richness. The common killifish inhabits coastal waters along the Atlantic coast of North America, typically living in dense shoals that may include thousands of individuals: they are oviparous and spawn from late spring to early fall, depending on latitude [28]. Notably, *F*. *heteroclitus* survive large temperature and salinity ranges [29] and appear to tolerate a wide range of contaminants (Burnett, 2007). Additionally, *F*. *heteroclitus* are omnivorous and act as a major prey source for fish-eating birds and other predatory fishes [30]. This trophic centrality has been suggested to be a driver of indirect life-cycle parasite persistence [31], and in other parasite community studies they have been found to host at least 22 species of helminth parasites [32]. In Georgia, USA, at least three species of metazoan parasite with indirect life-cycles have been described infecting *F*. *heteroclitus*. These include a trematode, *Lasiotocus minutus*, that has *F*. *heteroclitus* as the definitive host, molluscs as the first intermediate host, and fish or crustaceans as the second intermediate host [33]. A cestode, *Glossocercus caribaensis*, that has fish-eating birds as the definitive host, crustaceans as the first intermediate host, and *F*. *heteroclitus* as the second intermediate host [34]. The last indirect life-cycle parasite was a nematode, *Contraceacum sp*., that can parasitize fish-eating birds and mammals as definitive hosts, and *F*. *heteroclitus* as the sole intermediate host.

At each timepoint, 5 baited minnow traps were randomly placed at every site, during the flood tide and collected during the ebb tide. We placed all fish from the 5 traps into a holding tank, and randomly subsampled 30 killifish to obtain a representative population of each site and date combination. This sampling did not take into consideration the size or age of the fish, however prior research has suggested that a sample size of 30 from a host population is appropriate to capture parasite community diversity within a host population [35]. The fish from each collected site were separately housed in the animal facility at Georgia Southern University and necropsied within 1 week (IACUC #I14013).

Each fish was euthanized in a buffered 300 mg L^-1^ solution of tricaine methanesulfonate (MS-222) until cessation of opercula movement followed by sex determination and measurements of total length and weight. The MS-222 solution, all external surfaces, gills, and opercula were examined for ectoparasites and helminths. The viscera, gonads, spleen, liver, heart, intestine, and bladder were removed and examined for helminths. All helminths from the first collection were heat fixed, stored in 70% ethanol, stained in acetocarmine, and mounted in Permount. Parasites from all collections were identified with keys and primary literature [32,36].

### Statistical analyses

To visualize the variation in parasite component community and infracommunity structure, we performed non-metric multi-dimensional scaling (NMDS). In the NMDS for component communities, average abundances were calculated and square root transformed to control for the effect of high abundance parasite taxa. In the NMDS for infracommunities, raw abundances were ln(x + 1) transformed. Resemblance matrices were constructed using Bray-Curtis dissimilarity distance, and stress was calculated using Kruskal’s stress formula 1. To determine whether there were significant differences in parasite component community structure, we performed a permutational multivariate ANOVA (PerMANOVA). For this analysis, we used site as a factor and performed 999 permutations. Additionally, we performed a 2-way crossed similarity percentage (SIMPER) analysis to identify the parasite species that contributed to 5% or greater of the variation in parasite community structure among sites. These analyses were conducted in R statistical environment using package "vegan" [37,38].

To determine the roles of landscape composition and physicochemical factors in structuring the parasite infracommunity and component community, we built multivariate random forest (MRF) models using R package randomForestSRC [39]. MRF is an ensemble machine learning method, that builds a suite of bootstrapped multivariate regression trees [40]. MRFs are increasingly being used in ecological studies because they allow multivariate species composition response data, do not assume independence among samples, make no *a priori* assumptions on the relationships between the response and predictor variables, and can account for imbalanced data [41,42]. Our response variable was the multivariate parasite species data, and our predictor variables included 8 landscape factors (Table 1), 23 physicochemical concentrations (Tables 2 and 3), and host length, weight, and sex. We built 4 MRFs, examining landscape and physicochemical factors effects on direct and indirect parasite communities at the infracommunity and component community scale. MRFs were built using 1000 trees and "Breiman-Cutler" variable importance was calculated through permutation. Variable importance was standardized to average variable effect across species. In these variable importance permutations, each predictor variable is placed in the out-of-sample (out-of-bag for univariate analyses) data for the tree model. The out-of-sample prediction error is then calculated with and without the permutation and averaged over the MRF trees. Greater differences between the permuted and non-permuted out-of-sample prediction error equate to greater variable importance.

**Table 1:** Landscape characteristics of salt marsh sites.

	SK	SB	FP	CI	SM	TB
Total Area	8482	10441	47497	18101	11179	18101
Patch Density	39.6	29.9	24.2	23.9	28.2	23.9
Largest Patch Index	11.7	7.9	19.5	26.9	17.2	26.9
Edge Density	119.8	115.5	93.1	99.1	104.8	99.1
Mean Marsh Size	2.5	3.5	4.1	4.2	3.6	4.2
% Salt Marsh	39.2	25.6	3.2	46.7	33.2	46.7
% Developed	20.6	3.8	24.1	6.2	20.4	6.2
% Area Change to Developed (1996–2010)	6.4	1.9	18.5	2.6	7.3	2.6

The sites include: Fort Pulaski (FP), Cockspur Island (CI), Tybee (TB), and Skidaway Island (SK) in the Savannah River Estuary; Shellman Bluff (SB) as a representative of the Sapelo Sound; and St. Marys (SM) as part of the St. Marys Estuary. The variables measured reflect regional (HUC-12 digit watershed) characteristics of landscape heterogeneity and composition. These data were obtained from the National Landcover Dataset, and analyzed in FRAGSTATS and ArcGIS. The units of measure are acres for Total Area, # of land use class patches per watershed area for Patch Density, % of the landscape that the largest land use class patch comprises for Largest Patch Index, the number of edges between different land use classes per watershed area for Edge Density, and mean size of salt marsh patches for Mean Marsh Size. The sites are presented from left to right to reflect increasing levels of impervious surface within a 250 m radius.

**Table 2:** Mean metal concentration (mg/L) in water of salt marsh sites.

	Sites					
Metal	SB	SK	FP	CI	SM	TB
Ag	0.8 ± 0.4 **	0.8 ± 0.4 *	0.0 ± 0.0	0.1 ± 0.1	0.2 ± 0.1 **	0.3 ± 0.2
As	160.9 ± 33.2 **	149.9 ± 27.7 **	182.7 ± 42.5 **	143.4 ± 27.2 **	147.9 ± 27.8 **	153.6 ± 25.5 **
B	3229.6 ± 178.2 *	3289.1 ± 259.8 *	3022.7 ± 205.6 *	2921.7 ± 163.5 *	3039.5 ± 198.4 *	3607.1 ± 381.7 *
Ba	85.5 ± 15.8	93.4 ± 21.3	109.4 ± 25.4	109.6 ± 25.6 *	113.1 ± 26.1 *	113.5 ± 28.7 *
Co	3.7 ± 0.8 *	2.9 ± 0.6 *	4.4 ± 1.0 *	3.6 ± 0.9 *	3.2 ± 0.6 *	3.2 ± 0.6 *
Cu	65.1 ± 17.6 **	47.9 ± 11.7 **	52.2 ± 13.2 **	48.2 ± 12.0 **	48.1 ± 12.1 **	47.2 ± 11.1 **
Fe	909.0 ± 272.4 **	931.4 ± 274.0 **	1204.4 ± 282.7 **	1138.2 ± 318.8 **	1399.8 ± 450.4 **	1180.6 ± 334.6 **
Mn	74.8 ± 15.5 *	24.3 ± 7.3	101.6 ± 33.7 *	73.3 ± 36.6 *	69.1 ± 20.7 *	34.3 ± 10.1
Mo	34.6 ± 8.1 *	32.6 ± 7.3 *	34 ± 8.1 *	31.8 ± 7.5 *	33.9 ± 8.2 *	36.4 ± 8.1 *
Tl	28.3 ± 12.6 *	14.5 ± 5.2 *	20.6 ± 6 *	16 ± 5.6 *	18.3 ± 7.2 *	14.1 ± 4.2 *
V	349.9 ± 82.7 **	349.3 ± 80.0 **	357 ± 83.6 **	325.6 ± 75.3 **	328.7 ± 74.9 **	344.8 ± 77.1 **
Zn	68.1 ± 19.7 **	62 ± 17.9 **	76.9 ± 24.7 **	72.3 ± 23.0 **	49.1 ± 14.6 **	76.6 ± 24.2 **

The sites include: Fort Pulaski (FP), Cockspur Island (CI), Tybee (TB), and Skidaway Island (SK) in the Savannah River Estuary; Shellman Bluff (SB) as a representative of the Sapelo Sound; and St. Marys (SM) as part of the St. Marys River Estuary.

* represent mean concentrations containing at least one sample exceeding marine surface water guidelines for chronic toxicity, according to NOAA Screening Quick Reference Table guidelines [43].

** represents mean concentrations containing at least one sample exceeding marine surface water guidelines for acute toxicity, according to NOAA Screening Quick Reference Table guidelines.

**Table 3:** Mean metal concentration (mg/kg) in sediment of salt marsh sites.

	Site					
Metal	SB	SK	FP	CI	SM	TB
V	54.2 ± 4.5 *	42.3 ± 5.1	49.2 ± 3.9	52.2 ± 0.9	40.7 ± 3.5	29.4 ± 4.2
Cr	74.6 ± 6.8 **	37.7 ± 20.8	58.4 ± 1.9 *	47.9 ± 24 *	58.5 ± 6.6 *	46.8 ± 5.3
Co	9.7 ± 1.1 *	13.7 ± 2.7 *	6.8 ± 0.5	8.2 ± 0.3	8.0 ± 0.7	6.2 ± 0.4
Ni	45.7 ± 14.5 *	51.4 ± 12.5 *	5.6 ± 2.3	66.8 ± 32.7 *	80.2 ± 53.0 *	40.6 ± 13.3 *
Ag	0.8 ± 0.3 *	0.8 ± 0.3 *	0.4 ± 0.1	0.4 ± 0.2	0.6 ± 0.2 *	0.2 ± 0.0

The sites include: Fort Pulaski (FP), Cockspur Island (CI), Tybee (TB), and Skidaway Island (SK) in the Savannah River Estuary; Shellman Bluff (SB) as a representative of the Sapelo Sound; and St. Marys (SM) as part of the St. Marys River Estuary.

* represents mean sediment concentrations containing at least one sample exceeding the median toxic dose (T_50_) of marine benthic organisms, according to NOAA Screening Quick Reference Table guidelines [43].

** represents mean sediment concentrations containing all samples exceeding the median toxic dose (T_50_) of marine benthic organisms, according to NOAA Screening Quick Reference Table guidelines.

## Results

### Parasite community in *F*. *heteroclitus*

Eight taxa of metazoan parasites were identified from 630 fish, representing 24 different parasite communities. The direct life-cycle parasites included the branchiuran *Argulus funduli*, the copepod *Ergasilus funduli*, the leech *Myzobdella lugubris*, and the monogeneans *Swingleus ancistrus* and *Fundulotrema prolongis*. The indirect life-cycle parasites included the digenean *Lasiotocus minutus*, the cestode *Glossocercus caribaensis*, and the nematode genera *Contracaecum* sp. These 8 taxa of parasites infected more than 70% of the killifish examined (Tables 4 and 5).

**Table 4:** Prevalence (%) and mean intensity (SE) of the 3 indirect life-cycle parasite taxa infecting the salt marsh fish, *F*. *heteroclitus*.

	*Lasciotocus minutus*		*Glossocercus caribaensis*		*Contracaecum* sp.	
	Prevalence	Intensity	Prevalence	Intensity	Prevalence	Intensity
SK	26.7	14.0 ± 2.9	44.8	4.0 ± 0.6	1.0	1.0 ± 0.0
SB	24.8	24.6 ± 4.4	39.0	3.9 ± 0.5	7.6	1.0 ± 0.0
FP	22.9	9.5 ± 1.7	448	4.2 ± 0.5	13.3	1.0 ± 0.0
CI	2.9	7.3 ± 2.4	35.2	3.4 ± 0.6	5.7	1.0 ± 0.0
SM	1.9	4.0 ± 3.0	91.4	6.0 ± 0.5	6.7	1.0 ± 0.0
TB	0	-	30.5	2.2 ± 0.3	3.8	1.0 ± 0.0

The sites include: Fort Pulaski (FP), Cockspur Island (CI), Tybee (TB), and Skidaway Island (SK) in the Savannah River Estuary; Shellman Bluff (SB) as a representative of the Sapelo Sound; and St. Marys (SM) as part of the St. Marys Estuary. The sites are presented from top to bottom to reflect increasing levels of impervious surface.

**Table 5:** Prevalence and mean intensity (SE) of the 5 direct life-cycle parasite taxa infecting the salt marsh fish, *F*. *heteroclitus*.

	*Myzobdella lugubris*		*Swingleus ancistrus*		*Fundulotrema prolongis*		*Ergasilus funduli*		Argulus funduli	
	Prevalence	Intensity	Prevalence	Intensity	Prevalence	Intensity	Prevalence	Intensity	Prevalence	Intensity
SK	0	-	40.0	2.8 ± 0.4	29.5	4.6 ± 1.0	24.8	3.4 ± 0.7	15.2	1.4 ± 0.2
SB	1.9	1.0 ± 0	36.2	3.1 ± 0.3	20.0	4.2 ± 0.9	19.0	3.8 ± 0.7	20.0	1.4 ± 0.2
FP	9.5	1.2 ± 0.2	38.1	4.5 ± 0.7	29.5	6.0 ± 1.1	14.3	3.6 ± 0.7	24.8	1.4 ± 0.1
CI	0	-	8.6	2.0 ± 0.5	16.2	3.6 ± 0.8	20.0	3.9 ± 1.1	7.6	1.1 ± 0.2
SM	0	-	13.3	2.7 ± 0.5	20.0	3.6 ± 0.9	22.9	3.4 ± 0.9	12.4	1.2 ± 0.2
TB	0	-	16.2	1.4 ± 0.3	19.0	3.5 ± 0.5	11.4	3.1 ± 0.5	10.5	1.3 ± 0.1

The sites include: Fort Pulaski (FP), Cockspur Island (CI), Tybee (TB), and Skidaway Island (SK) in the Savannah River Estuary; Shellman Bluff (SB) as a representative of the Sapelo Sound; and St. Marys (SM) as part of the St. Marys Estuary. The sites are presented from top to bottom to reflect increasing levels of impervious surface.

**Fig 2:**
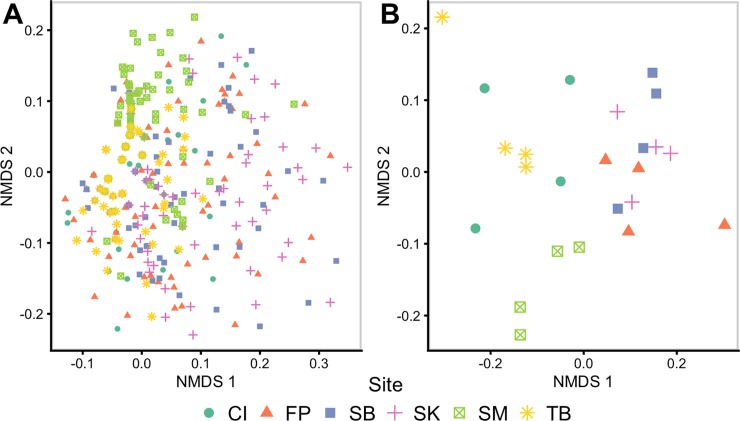
**Non-metric multidimensional scaling plots of parasite infracommunities (A) and component communities (B) of the salt marsh fish, *F*. *heteroclitus*, from 6 sites in coastal Georgia, USA.** Infracommunity (A) distances are based on Bray-Curtis dissimilarities of the ln(x +1) transformed abundances of 8 parasite taxa. Component community (B) distances are based on Bray-Curtis dissimilarities of the square-root transformed average abundances of 8 parasite taxa. The sites include: Fort Pulaski (FP) (salmon triangles), Cockspur Island (CI) (aquamarine circles), Tybee (TB) (yellow stars), and Skidaway Island (SK) (pink crosses) in the Savannah River Estuary; Shellman Bluff (SB) (blue squares) as a representative of the Sapelo Sound; and St. Marys (SM) (green crossed squares) as part of the St. Marys Estuary.

Assessed with NMDS and PerMANOVA, infracommunity composition differed among sites (*P* < 0.001, Pseudo F_5,629_ = 15.691; Fig 2A). Additionally, the parasite component community composition differed among sites (*P* < 0.001, Pseudo F_5,12_ = 6.11; Fig 2B). SIMPER analysis indicated that the observed differences in parasite community structure were driven by indirect life-cycle parasites: the digenean *Lasciotocus minutus*, and the cestode *Glossocercus caribaensis*. This pattern was consistent when considering the parasite component community and the parasite infracommunity. This analysis demonstrated that direct life-cycle parasites did not contribute to the variation in parasite community structure.

### Landscape and physicochemical predictors of parasite community in *F*. *heteroclitus*

**Fig 3:**
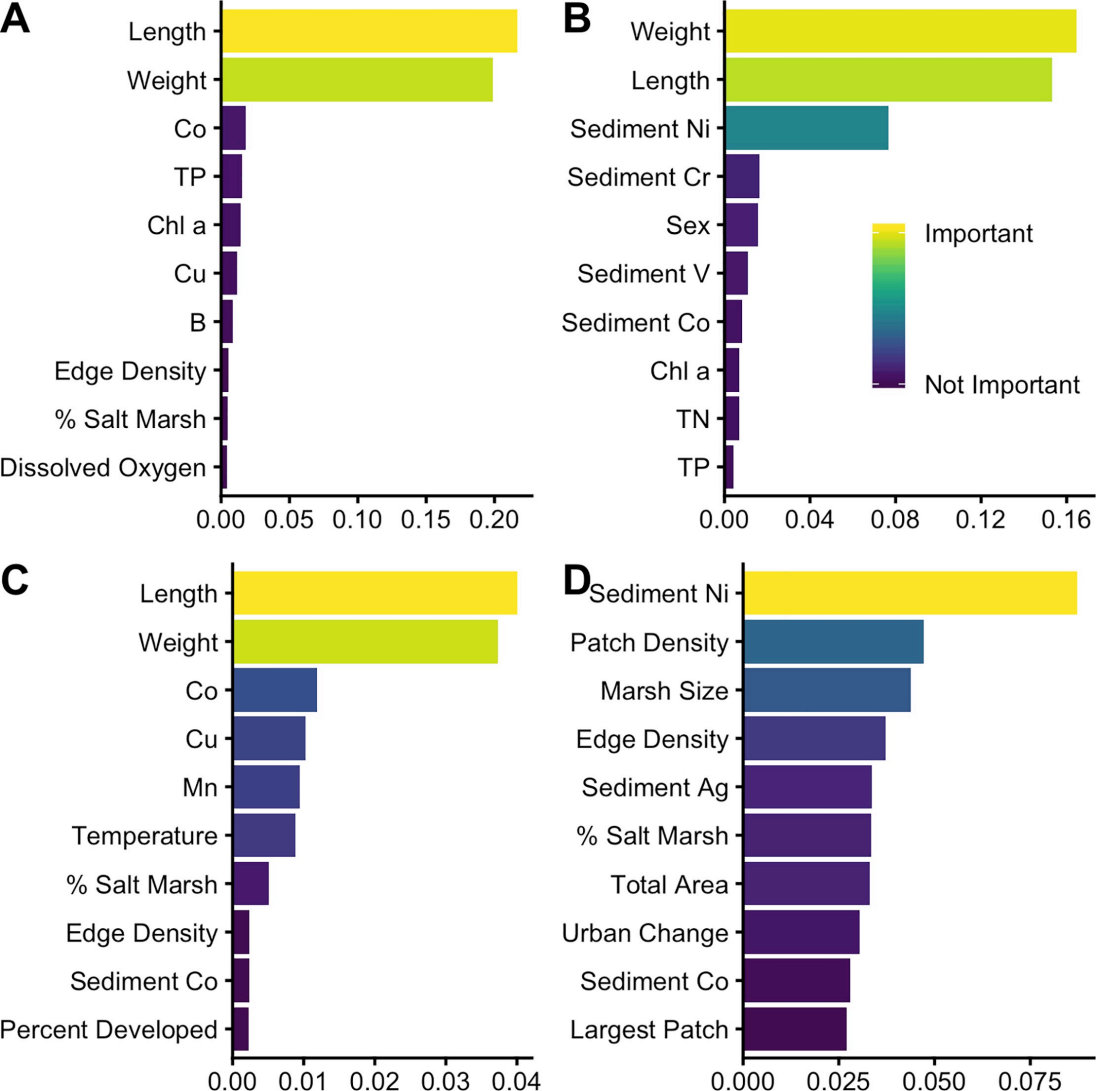
Multivariate random forest model standardized variable importance. Variable importance in the model predicting: direct life-cycle parasite community structure at the infracommunity scale (A); indirect life-cycle parasite community structure at the infracommunity scale (B); direct life-cycle parasite community structure at the component community scale (C); and indirect life-cycle parasite community structure at the component community scale (D).

We identified 12 metals from water analyses and 5 metals from sediment analyses that surpassed concentration thresholds (Tables 2 and 3), and our spatial analysis included 9 landscape metrics that varied across our collection sites (Table 1). We built MRF models to determine important variables associated with direct and indirect life-cycle parasites at the infracommunity (within an individual host) and component community (within a host population at a collection site) scales. The model predicting direct life-cycle parasite community structure at the infracommunity scale explained 40.18% variance, with fish weight and length being the most important variables (Fig 3A). The model predicting indirect life-cycle parasite community structure at the infracommunity scale explained 21.4% variance, with fish weight, fish length, and percent change in developed land being the most important variables (Fig 3B). The model predicting direct life-cycle parasite community structure at the component community scale explained 70.67% variance, with fish weight and length being the most important predictors (Fig 3C). The model predicting indirect life-cycle parasite community structure at the component community scale explained 73.43% variance, with sediment Ni, patch density, and marsh size being the most important predictors (Fig 3D).

## Discussion

The goal of this study was to determine the effects of urbanization, mediated through alterations in landscape composition and physicochemical factors, in structuring parasite communities. Our sampling in coastal Georgia, USA provided evidence supporting the role of urbanization in impacting salt marshes via increased metal contamination, altering landscape composition at a fine spatial scale (250 m), and at the watershed level. We determined that parasite community composition differed among sites with different levels of urbanization. Variation in the prevalence and intensity of infection by indirect life-cycle parasites, the digenean *L*. *minutus* and the cestode *G*. *caribaensis*, were the primary drivers for the differences in parasite communities. Although the prevalence of direct life-cycle parasites differed among sites, this difference did not contribute to overall variation in component community composition. We found that factors driving community structure differed between the infracommunity and component community for indirect life-cycle parasites. Host size was the most important variable for direct life-cycle parasite assemblages and indirect life-cycle parasites at the infracommunity level, while landscape and physicochemical factors determined the structure of indirect life-cycle parasite assemblages at the component community scale. These data support the prediction that in the common marsh killifish, parasite community composition is explained by metrics associated with human modification of the environment, and parasite responses to urbanization are dependent upon life history.

Our results indicate that anthropogenic physicochemical and landscape factors structure indirect life-cycle parasites at the component community scale. The most important physicochemical factor was sediment nickel concentration, and although nickel occurs naturally and is essential to most organisms as a trace mineral, increased concentrations due to human activities (i.e., burning fuel [44]) can be toxic to salt marsh benthic organisms. For example, Field et al. (2002) built a suite of logistic regression models based on toxicity assays of marine amphipods, and predicted that 50% of the population would show toxicity effects at concentrations of 47 ppm [45]. The concentrations we observed exceed this level, and may affect intermediate host populations that are an integral part of the transmission of the indirect life-cycle parasites we described in our study, the digenean *Lasiotocus minutus* and the cestode *Glossocercus caribaensis*. Additionally, these indirect life-cycle parasites have life stages that are external to the host, and the nickel concentrations we measure may be toxic to these free-living parasite life stages further impacting parasite component community diversity.

The most important landscape factors for indirect parasites at the component community level were marsh size and patch density. Marsh size has been shown to affect carbon pathways to resident invertebrates, suggesting potential impacts on animal community and resultant trophic structure and interactions that are exploited by indirect life-cycle parasites for transmission [46]. For example, Lowe and Peterson (2014) found marsh size to be positively correlated with abundances of Gulf Menhaden and brown shrimp, though they did not find an association with blue crab abundance in the same sites[1]. Patch density is a measure for landscape heterogeneity. In urbanized estuaries, salt marshes become increasingly fragmented and the landscape increases in heterogeneity [47]. This often has the result of isolating habitat patches, leading to alterations in animal community structure and trophic interactions [48,49]. For example, Rudershausen (2018) found that salt marsh connectivity influenced fish community structure and the probability of *F*. *heteroclitus* movement [50]. These studies illustrate that landscape alterations have the potential to alter free-living host community structure; given that indirect life-cycle parasites require stable trophic interactions [5] between animals across trophic levels, altering the structure of the free-living community will necessarily be reflected in the parasite community [51].

Although the infracommunity is a sample within the component community, we found the factors that structure indirect life-cycle parasites differ between these two levels. Parasite transmission is inherently a local phenomenon, and individual risk factors do not necessarily equate to population risk factors. For example, the encounter-dilution effect hypothesizes that individuals can decrease their individual risk of parasitic infection through the formation of groups [52] even as the population level of infection increases. We found that the diversity of parasites in an individual fish was driven by host size (length and weight), but the diversity of parasites in the population was mostly affected by abiotic measures (sediment Ni, patch density, and marsh size). This subtle difference is because parasite community assembly is influenced by processes operating at a range of spatial and temporal scales. Parasite species are found within a regional species pool that is constrained by evolutionary processes, and only a subset of the species from the regional pool will colonize a site depending on ecological factors such as dispersal and the probability of encountering a suitable host. Subsequently, a subset of the species from the regional pool will colonize a particular site depending on dispersal and exposure probability. In essence, our data suggests that the observed parasite community within a host and the factors that determine what parasites you find in that host are dependent on the scale at which the study is conducted because of different abiotic and biotic filters [20,51,53,54].

Finally, our results indicate that host body size was the strongest determinant of direct life-cycle parasites at the infracommunity and component community scales. Individual hosts can be thought as habitat "islands" for parasites [55]. For direct life-cycle parasites, island biogeography theory can be applied [56], wherein larger hosts are larger islands, and consequently provide a larger habitat for a parasite to colonize, allowing for a greater number of parasite individuals and concurrent coexisting species [57,58]. In fish, body growth is largely indeterminate, and is associated with age, which may result in older fish harboring more parasite species and individuals simply because of an increase in the opportunity for acquiring an infection.

Previous studies have shown a relationship between urbanization and parasite community variation. Blanar et al. (2016) demonstrated a correlation between parasite community structure and local contaminants and land use within 5 km [9]. In this study, the relative abundance of the indirect life-cycle digenean was positively associated with an increase in crude oil contaminants, whereas the opposite pattern was observed for the directly transmitted monogenean parasites. This observation follows results of an earlier meta-analysis, where parasite life history (>1 obligatory host in life-cycle vs. 1 obligatory host in life-cycle) and habitat (external vs. internal) informed whether aquatic pollution had a significant effect on parasite population dynamics [59]. Additionally, Calegaro-Marques and Amato (2014) revealed an association between an urban-rural gradient and life-cycle variation in parasite communities of Rufous-bellied thrushes [8]. Our results agree with these studies, and support the proposition that urbanization disproportionately affects indirect life-cycle parasites, which is then reflected in the parasite component community structure.

Coastal urbanization and anthropogenic disturbance are rapidly altering free-living and parasite community dynamics. Our results suggest that urbanization and its effects are important in structuring parasite communities of the common marsh killifish, indirect life-cycle parasites are more affected than direct life-cycle parasites, and the degree to which individual parasite species respond to urbanization is determined by their specific life history strategy. Further, our data suggest that urbanization likely affects interactions of free-living hosts, and their environment which is then detected as variation in the parasite community.
